# High-Temperature and Drought-Resilience Traits among Interspecific Chromosome Substitution Lines for Genetic Improvement of Upland Cotton

**DOI:** 10.3390/plants9121747

**Published:** 2020-12-10

**Authors:** Kambham Raja Reddy, Raju Bheemanahalli, Sukumar Saha, Kulvir Singh, Suresh B. Lokhande, Bandara Gajanayake, John J. Read, Johnie N. Jenkins, Dwaine A. Raska, Luis M. De Santiago, Amanda M. Hulse-Kemp, Robert N. Vaughn, David M. Stelly

**Affiliations:** 1 Department of Plant and Soil Sciences, Mississippi State University, Mississippi State, MS 39762, USA; rajubr@pss.msstate.edu (R.B.); kulvir@pau.edu (K.S.); lokhande.suresh.bajirao@gmail.com (S.B.L.); gajawyb@yahoo.com (B.G.); 2 USDA-ARS, Genetics and Sustainable Agriculture Research Unit, Mississippi State, MS 39762, USA; john.read@usda.gov (J.J.R.); johnie.jenkins@usda.gov (J.N.J.); 3 Department of Soil and Crop Sciences, Texas A&M AgriLife Research, College Station, TX 77843, USA; minorcat123@yahoo.com (D.A.R.); luis.desantiago@austin.utexas.edu (L.M.D.S.); amanda.hulse-kemp@usda.gov (A.M.H.-K.); bobv@tamu.edu (R.N.V.); stelly@tamu.edu (D.M.S.); 4 USDA-ARS, Genomics and Bioinformatics Research Unit, Raleigh, NC 27695, USA

**Keywords:** abiotic stress tolerance, crop physiology, gas exchange, germplasm utilization, global warming, heat stress, sustainability

## Abstract

Upland cotton (*Gossypium hirsutum* L.) growth and development during the pre-and post-flowering stages are susceptible to high temperature and drought. We report the field-based characterization of multiple morpho-physiological and reproductive stress resilience traits in 11 interspecific chromosome substitution (CS) lines isogenic to each other and the inbred *G. hirsutum* line TM-1. Significant genetic variability was detected (*p* < 0.001) in multiple traits in CS lines carrying chromosomes and chromosome segments from CS-B (*G. barbadense*) and CS-T (*G. tomentosum*). Line CS-T15sh had a positive effect on photosynthesis (13%), stomatal conductance (33%), and transpiration (24%), and a canopy 6.8 °C cooler than TM-1. The average pollen germination was approximately 8% greater among the CS-B than CS-T lines. Based on the stress response index, three CS lines are identified as heat- and drought-tolerant (CS-T07, CS-B15sh, and CS-B18). The three lines demonstrated enhanced photosynthesis (14%), stomatal conductance (29%), transpiration (13%), and pollen germination (23.6%) compared to TM-1 under field conditions, i.e., traits that would expectedly enhance performance in stressful environments. The generated phenotypic data and stress-tolerance indices on novel CS lines, along with phenotypic methods, would help in developing new cultivars with improved resilience to the effects of global warming.

## 1. Introduction

Upland cotton (*Gossypium hirsutum* L.) is one of the most important cash crops in the United States and worldwide. Although Upland cotton is relatively heat- and drought-resilient compared to some other major crops, enhancements are needed to maintain production, further improve sustainability, and confront global climate change. Most of the cotton-producing regions frequently face shortages of irrigation water due to variable rainfall and access to irrigation or irrigation water, commonly coupled with higher evapotranspiration demands [1,2]. The global surface temperature has increased by approximately 0.6–0.85 °C since 1880 and is projected to continue, increasing 0.2 °C per decade [3]. In addition to decreasing and uneven precipitation, warmer days with a decreased diurnal temperature incidence are projected to occur more often in the future [4,5]. A combination of too high temperatures and drought events could reduce major food crops’ yields up to 60% worldwide ([6,7]. Furthermore, a comparison of reported average crop yields with historical record yields in the USA-grown crops is one third to one seventh of record, due to unfavorable environmental conditions [8].

Due to the indeterminate growth habits of cotton, the stored soil moisture and local microclimatic conditions strongly influence new branching nodes, flowers, bolls, and the partitioning of resources between source and sink [1,2]. In the southern United States, reproductive and boll formation stages of cotton coincide with high temperatures and low precipitation. A rise in temperature above the optimum (>30 °C) in July-August combined with high humidity and low rainfall results in significant impairment to the plant growth of cotton, changes leaf biochemical and biophysical properties and reduces boll formation and boll size due to higher evapotranspiration demand under field conditions [2,9,10,11]. While small variations in temperature may not cause significant damage to seed weight/boll weight, such a narrow spike in temperatures during anther dehiscence causes a substantial reduction in fertility/seed number ([12,13,14]. Short periods of heat events (>30 °C) are reported to significantly limit cotton productivity by disrupting a series of reproductive events [12,15,16]. Frequent heat spikes during flowering impair reproduction by inhibiting male [17] and female [18] gametophyte development, pollen germination [19], and pollen tube length growth [20]. Similar to other crops, a decline in boll set and the abortion of squares was observed upon exposure to daytime temperatures exceeding 30 °C for about 13 h both in Upland and Pima cotton [2,12,21,22,23].

Genetic variability in physiological, reproductive traits, lint yield, and fiber quality in cotton is evident in cotton, indicating the potential to increase stress tolerance [24,25]. To date, selections have been focused on yield and fiber quality traits to develop progenitors of *G. hirsutum* from its wild relatives [26] for non-stress conditions. Wild resources have been utilized in the breeding program to improve the stress resilience of Upland cotton lines by stacking progenitor alleles. G. *barbadense* (superior fiber quality) and *G. tomentosum* (heat-resistant species of the genus, [27] have been extensively used to explore beneficial alleles for useful traits in the breeding programs [26,28,29,30,31,32]. For example, *G. barbadense* (Pima cotton) is being used as a donor for fiber quality [26,33]. However, even with the continued genetic gain (2015–2019), lint yields of Upland cotton average 600 kg ha^−1^ more than Pima cotton [34]. This difference in cotton yield could be due to differences in stress tolerance [12,13,35].

High temperature and drought trigger morph-physiological, biochemical, and molecular alterations leading to weaker plant growth and productivity, and ultimately yield in many crops, including cotton [35,36]. Among numerous traits, plants’ ability to maintain robust growth and development under stress signifies the greater carboxylation efficiency, a cooler canopy, and greater enzyme activities at mitochondria and chloroplasts (high chlorophyll and membrane stability). The impacts of heat and drought stress on morphological (leaf area and biomass), physiological (gas exchange and pigments), biophysical (membrane fluidity or stability and canopy temperature), and reproductive (pollen germination ability) traits have been widely studied across crops [37,38,39]. For instance, gas exchange (photosynthesis and stomatal conductance), canopy temperature, and cellular or membrane stability traits are integrated attributes that reflect the morphological, anatomical, physiological, and biochemical features at the root and leaf [36,37,38,39]. Genetic variability in these traits has been effectively utilized to understand mechanisms, discriminate between tolerant and sensitive genotypes, and for crop improvement under different stress conditions [37,38,39].

The effects of these abiotic stresses are often inter-related. Previous analyses of CS lines have associated many novel traits with substituted chromosomes and chromosome segments from the wild species and unadapted germplasm [29,30,31,32]. However, the existing collection of CS lines have not been tested for physiological and reproductive traits associated with enhanced agronomic performance under field conditions. The long-term goal of screening cotton breeding lines and new germplasm for abiotic stress tolerance, such as heat and drought, is to identify a new genetic variation that minimizes yield losses in common cotton-producing environments. Genetic gains would complement farm management efforts that partially alleviate stress effects and stabilize cotton yields across increasingly variable environmental conditions. Given that genomes of CS lines are mostly of Upland origin and true-breeding, CS lines identified as stress-resilient can be directly incorporated into breeding programs aiming to improve sustainability and guard against the climate crisis, which has significantly projected more frequent episodes of higher temperatures and drought intensities. This research’s immediate objective was to identify cotton substitution lines with improved traits against the two most important challenges (high temperature and drought) in sustainable cotton production.

We utilized CS lines developed through hypoaneuploidy-based backcrossing, using “TM-1” as a recurrent parent, to quantify selected physiological and reproductive characteristics associated with line yield. The present study investigated CS lines’ performance in carrying a wild genomic composition of *G*. *barbadense* and *G. tomentosum* in TM-1 background for leaf gas exchange, pigments, biophysical and reproductive traits under field conditions. We classified the CS lines based on the degree of tolerance to heat and drought using vegetative, physiological, and reproductive parameters. Finally, we associate the assessed phenotype with exotic substitution chromosome/s to identify potentially favorable CS lines for future cotton breeding.

## 2. Results and Discussion

The present study characterized CS lines under field conditions to identify potential CS lines with differences in leaf gas exchange, pigments, biophysical and reproductive traits compared with TM-1. Each CS-B or CS-T line was bred to replace a pair of chromosomes or chromosome arms of *G. hirsutum* inbred TM-1, with the respective pair from *G. barbadense* doubled-haploid 3–79 or *G. tomentosum*, respectively [28,31,40]. These backcrossed CS lines are nearly isogenic to the recurrent parent TM-1 for 25 chromosome pairs and similar to each other for 24 chromosome pairs [26,28]. Therefore, this uniform genetic background provides us with opportunities for comparing these lines and an accentuated possibility of detecting a genetic effect(s) of the substituted chromosomes and chromosome arms on specific traits inherited from the alien species.

**Figure 1:**
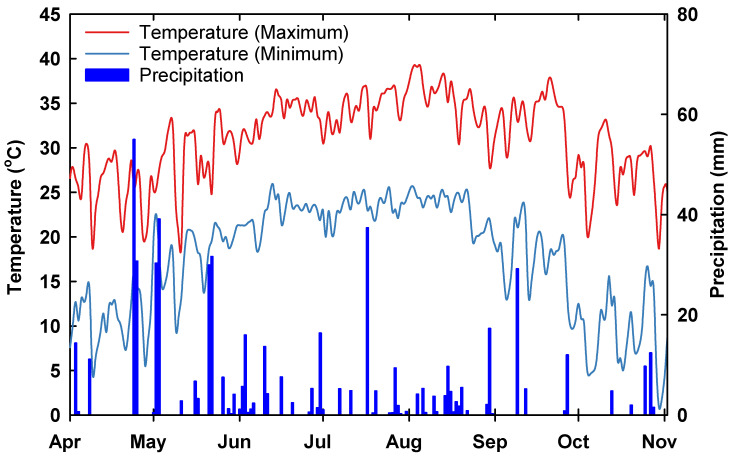
Daily weather parameters (temperature and rainfall) during crop growing season. Data were obtained from the *PRISM weather* data (http://www.prism.oregonstate.edu) for the experiment site.

Planting was done to coincide with the reproductive stage of the CS lines to occur specifically between late-July and mid-August. The range in air temperature (minimum and maximum) and rainfall during the cropping season is visualized in Figure 1. We observed the maximum air temperature combined with the lowest rainfall at the experiment site during this period. The average maximum air temperature varied from 34.4 °C (SD ± 1.9) in July to 35.3 °C (SD ± 2.8) (Figure 1), which is 4.4 to 5.3 °C above the optimum temperature of 30 °C for successful growth, development and reproduction in cotton [2,12,21,22,23]. On the other hand, on nearly all days in July (30 days) and August (29 days), the maximum daily temperature was above 30 °C. The same hotter window coincided with the year’s lowest rainfall; on average, about 58.5% lower rainfall was witnessed compared to the early vegetative stage (157.4 mm) (see Figure 1). The corresponding total rainfall was 68.7 mm in July and 63.7 mm in August. In the southern United States, a rise in temperature above the optimum (>30 °C) in July-August combined with low rainfall is known to induce damage to leaf gas exchange, biochemical and biophysical properties, and reduces boll formation and boll size due to higher evapotranspiration demand under field conditions [2,9,10,11]. In this study, CS lines differed significantly for many measuresd physiological and reproductive characteristics, and various response indexes (Table 1).

The relative performance of CS lines compared with TM-1, the recurrent parent of the CS lines, has been given in Table S1. The Dunnett’s test was performed using trait means to test the difference between each CS line and TM-1 and the overall result from the stress response index analysis for different traits used to identify CS lines that exhibit increased tolerance to high temperature, drought, or a combination of both abiotic stresses (Table 2).

### 2.1. Leaf Gas Exchange and Pigment Parameters

The eleven CS-B and CS-T lines varied significantly for various leaf gas exchange and pigment parameters (Table 1). Five CS lines had photosynthesis (Pn) significantly greater than TM-1, 13.7% on average (Table 1). These included three CS-T lines, namely CS-T07, CS-T18, and CS-T15sh, and two CS-B lines, CS-B04 and CS-B15sh (Table 2). Significant variation also occurred for stomatal conductance (Gs, mmol m^−2^ s^−1^). Two CS-T lines, CS-T07 and CS-T15sh, and one CS-B line, CS-B15sh, exhibited significantly greater Gs than TM-1, whereas one was considerably lower, i.e., CS-T04 (Table 2). Somewhat in parallel, lines CS-T15sh and CS-T04 also exhibited the maximum and minimum transpiration rates (T, mmol H_2_O m^−2^ s^−1^); the T for CS-T15sh (24%) was significantly higher than TM-1 (Table 1 and Table S1), and the T for CS-T04 was significantly lower than the average of all CS lines (11.48). In this study, Pn had significant correlations with Gs (*r* = 0.88, *p* < 0.001) and T (*r* = 0.86, *p* < 0.001), indicating the strong influence of stomatal conductance to CO_2_ on final leaf photosynthesis [42,43]. Thus, two CS lines (CS-T15sh and CS-T07) with increased Gs and T could be used as potential donors for improving heat tolerance (an indication of evaporative cooling) or cotton yield potential in a warmer environment [44,45].

**Figure 2:**
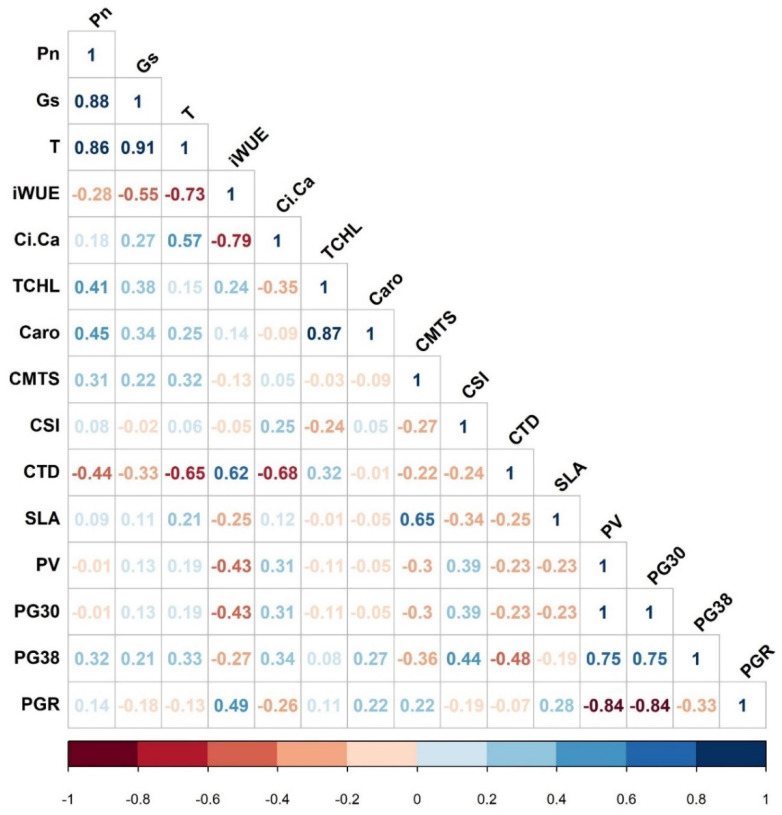
Cotton chromosome substitution (CS) line trait correlations of the recorded parameters. Dark color values represent strong correlations, and light color values represent weaker correlations. Values close to zero indicate no correlation between the two variables; whereas, values close to ± indicate strong correlation (positive—dark blue and negative-dark red) between two traits. Leaf gas exchange (Pn—photosynthesis, Gs—stomatal conductance, T—transpiration, Ci/Ca—leaf internal to ambient carbon dioxide concentration ratio and iWUE—instantaneous water use efficiency), cell and chlorophyll stability indices (CMTS—cell membrane stability index, and CSI chlorophyll stability index), biophysical (CTD—canopy temperature depression, and SLA—specific leaf area), and reproductive (PV—pollen viability, PG30—pollen germination at 30 °C and PGR—pollen germination response). Correlation values ≥ ±0.55 are significant at *p* < 0.05.

The water use efficiency of several crops is linked with Pn, T, or both [46]. Values of instantaneous water-use efficiency (iWUE) varied by a factor of 1.2 among the CS lines, with CS-T04 and CS-T15sh being the highest and lowest, respectively (Table 1). The iWUE of CS-T04 was significantly higher (16.6%) than the iWUE of TM-1, while CS-T15sh was nearly significantly lower than TM-1 (Table 1 and Table S1). The iWUE was negatively correlated with Pn, Gs, and T, with intensities increasing in that order (Figure 2). The strongest negative correlation for iWUE, however, was with Ci/Ca (*r* = −0.79, *p* < 0.001) (Figure 2). The Ci/Ca ratio was fairly strongly correlated with T (*r* = 0.57) (Figure 2). These results seem to suggest that the final carbon assimilation in CS lines was most strongly influenced by Gs. A higher iWUE in CS-T04 indicates a lesser Gs. Though CS-T07 and CS-T18 had a combination of high Pn and Gs, the iWUEs of these are not significantly different compared to TM-1 (Table 2). That variation among the CS lines for gas exchange traits was statistically significant, which suggests that genetic differences among the CS lines affected these traits.

Genetic difference in total chlorophyll content (TCHL) at the reproductive stage was significant at *p* < 0.05, and no CS line had greater TCHL than either TM-1 or the CS line average (45.6) for this trait (Table 1). Nonetheless, the fact that CS-T07 maintained greater TCHL (13%) and Pn (19%) at flowering compared to CS-B07 could reflect a differential genetic ability of CS-T07 to maintain chlorophyll content and higher photosynthetic rate (Table 1). Similarly, soybean with higher chlorophyll content showed a higher Pn at different growth stages [47]. During the reproductive phase in the current work, carotenoids (Caro, µg cm^−2^) showed a narrow range from about 9.3 to 11.0 and no significant variance due to differences among the lines (Table 1). However, there was a positive (*r* = 0.87) correlation between Caro and THCL (Figure 2). Because a plant with relatively high Pn value is associated with delayed senescence (or the ‘stay trait’), the CS-T07 line with greater leaf pigment values can reflect more efficient Pn as well as light-harvesting capacity, similar to studies in other species [48,49]. The combination of a relatively high Pn and TCHL (greener canopy at flowering) observed for CS-T07 and CS-T18 (Table 1) could indicate that these lines have a delayed senescence stay-green trait for improving agriculturally elite types of cotton.

### 2.2. Biophysical and Temperature-Tolerant Indices

Measurements of canopy temperature depression (CTD, °C) reported the difference between ambient air and canopy temperatures. They indicated the ability of a line to lower canopy temperature through transpiration-based cooling. Variation of CTD among CS lines was significant and ranged from −2.52 to 5.05, with three CS lines being significantly cooler than TM-1, namely CS-T15sh, CS-B04, and CS-T08sh (Table 1 and Table 2). CS-T04 had the hottest canopy, but differences from TM-1 were nonsignificant. The significance of internal leaf cooling in association with Gs driven by T and yield while under stress has been highlighted in *G. barbadense* Pima cotton [44]. Values for CTD were correlated negatively with transpiration (T) (*r* = −0.65, *p* < 0.05), and positively with iWUE (*r* = 0.62, *p* < 0.05) (Figure 2). Studies in Arizona on the impact of high temperature on cotton yields indicated that tolerant cultivars transpired more water, which resulted in cooler canopies that led to flowers and young bolls being less exposed to high temperatures [45,50]. In those studies, the high-temperature tolerant cultivars produced a greater yield than temperature-sensitive cultivars. Breeding cultivars tolerant to high- and low temperatures would be an alternative approach to minimize future cotton production’s negative impact. Low-temperature tolerance would enable farmers to plant early. Thus, flowering could occur before the mid-summer high temperatures that often limit the fruit set [51].

The resilience of cell membranes to elevated temperatures and greater chlorophyll stability values are correlated with heat and drought tolerance in cotton [41]. A higher cell membrane and chlorophyll stability value signifies a CS line plant’s ability to withstand stress due to greater cellular level tolerance, which would lead to increased photosynthesis, more dry matter production, and improved yield performance [52]. The variance of cell membrane temperature stability (CMTS) due to line effects was highly significant. While the average of CS lines (39.5%) was similar to that of TM-1 (39.8%), the values of the three CS lines, CS-B04, CS-T01, and CS-T18, were significantly higher than TM-1, and the importance of one line, CS-T08sh, was substantially lower than TM-1 (Table 1 and Table 2). Plants with high CMTS were used as a proxy to select high yielding cotton under high-temperature conditions [53]. While CMTS and CTD were slightly negatively correlated overall (Figure 2), CS-T04 exhibited the highest CMTS and the highest CTD (Table 1), indicating the existence of complex genetic interactions. The CSI is related to cotton plant tolerance to environmental stresses [41] and differed significantly among CS lines (Table 2).

Interestingly, none of the tested CS lines achieved greater CSI than TM-1, and the average CSI of CS lines (86.4%) was well below the observed TM-1 value (94%). While CSI values need not be consistent across studies, the present study’s values were within the range reported for 38 cotton cultivars [41], whereas the TM-1 value was spuriously high. Among all CS lines, CS-B04 had relatively high CMTS and CSI, the second lowest CTD, and among the highest Pn and T (Table 1).

Specific leaf area (SLA, cm^2^ g^−1^) is a critical plant functional trait, and variation in SLA is a useful indicator for leaf mass allocation under stress conditions [54,55]. The SLA values varied significantly and ranged from 117 cm^2^ g^−1^ in CS-B01 to 138 cm^2^ g^−1^ in CS-T01, with an average of 126 cm^2^ g^−1^ (Table 1). As compared to TM-1, CS-T01 recorded 7% greater SLA, whereas CB-B01 recorded 9.3% lower SLA (Table 1 and Table S1). SLA at the reproductive stage was most closely correlated with CMTS (*r* = 0.65) (Figure 2) and indicated that CS lines with high SLA might also maintain high CMTS under non-stressed conditions. In previous studies, low SLA has been used as an indicator of drought-tolerant lines in field crops [56,57,58] and in cotton [45] because thicker leaves (low SLA with high chlorophyll density) usually maintain higher photosynthetic capacity and WUE [59]. Thus, CS-T07, which had low SLA, high TCHL, and the highest Pn capacity, would seem to be prospectively useful as a source for drought tolerance attributes (Table S1).

### 2.3. Pollen Viability and Germination Parameters

Compromised pollen viability and function have been implicated as yield-influencing casualties of high temperatures in cotton [19] and other crops [60]. In this study, line-to-line differences significantly affected all of the pollen performance traits (Table 1). Pollen viability (PV), based on TTC staining, ranged from 33% in CS-T04 to 52% in CS-B07, with an average of 45%, roughly 80% of the observed TM-1 percentage (55%). Likewise, pollen germination (PG30) of CS lines at 30 °C was approximately lower by 20% than TM-1 (52%). However, CS-T15sh recorded high pollen germination (49%) at 30 °C, similar to TM-1 (Table 2). For all lines, the frequencies of PG30 were just a few percent lower than the PV rates, and patterns of variation among lines for PV and PG30 were nearly identical, resulting in a near-perfect correlation (Table 1 and Figure 2). Almost all of the pollens judged to be viable were capable of germination at 30 °C (non-stress condition). Five CS-lines had negative/lower pollen viability and germination percentages than the adapted Upland TM-1 cotton line (Table 2). The pollen viability and germination rates observed in the present study are similar to or slightly higher than observed previously in cultivars with similar methods [19,41] but lower than those observed using different methods [61]. The observed lower PV by 25% and PG by 27% (at 30 °C) compared to TM-1 in the present study might indicate stronger reproductive incompatibility, which could be due to interspecific and conspecific competitions between TM-1 and wild progenitors. These observations promote follow up studies to unravel the mystery of reproductive incompatibility between wild accessions and adapted cotton lines.

It is interesting to note that chromosomes 1, 4, and 18 from CS-B and CS-T lines in our previous studies generally showed a negative additive effect on lint yield in cultivars [29,30]. However, this report showed that chromosomes 1 and 4 from CS-B lines showed more significant additive effects in fiber quality traits than fiber strength in homologs. A previous study conducted in controlled-temperature sunlit plant growth chambers at the flowering stage showed that when Upland cotton plants were placed at 29 °C (long-term July temperature at Starkville, Mississippi, the USA plus 2 °C), all the flowers that were set after 5 days of stress gradually declined [51]. On the contrary, all the bolls abscised within five days after flowering in the sunlit plant growth chambers kept at the long-term July mean temperature plus 7 °C. About 200 to 250 bolls m^−2^ were retained in all temperatures; however, the number of bolls retained was strongly influenced by temperature. These observations suggest a need for follow up investigation to unravel the mystery of reproductive incompatibility at different temperatures between wild accessions and adopted Upland cotton lines and its effects on yield.

Production of sterile pollen under high temperatures has been associated with the early degeneration of the anther tapetal layer [62]. High day temperatures that exceeded 33 °C during heat-sensitive micro and macro sporogenesis resulted in reduced pollen viability and pollen number in many field crops [60]. Along the same line, decreased pollen viability could significantly affect pollen germination on the stigma [63,64], depending on the growth temperature [65]. Therefore, pollen germination inhibition under heat stress [64,66,67,68,69] negatively impacts the seed/fruit set in crops [69,70,71]. Upland cotton lines have been bred for heat tolerance by selecting progenies developed from surviving pollen grains pre-exposed to 35 °C for 15 min [72]. This result suggests that higher pollen viability germination could be used as a proxy to screen cotton cultivars for high-temperature tolerance. Burke et al. [61] recorded sharp declines in the percentages of cotton pollen germination at 37 (71%) to 40 °C (23%) and little germination at 43 °C. In this study, pollen grains showed a significant (*p* ≤ 0.001) decrease (23.1%) in the pollen germination percentage at 38 °C compared to 30 °C (Table 1). The PG38 for the CS lines varied by only about 11% in absolute terms, from nearly 27 or 28% for CS-T04 and CS-T01 to nearly 37 or 38% for CS-B04 and CS-T08sh. Thus, no line had substantially different PG38 than TM-1 (33.1%) (Table 1). While the overall line-to-line variance PG38 was significant statistically, most differences between individual lines and TM-1 were small relative to the LSD value of 5.9% and statistically significant for CS-T04 only (*p* < 0.05). The largest decrease in the pollen germination response (PGR) to elevated temperature was recorded in TM-1 (37%). However, this merely followed the overall trend that the variation in PGR values among lines largely reflected the variation in PG30 (*r* = −0.84), rather than the variation in PG38 (*r* = −0.32) (Figure 2). Additional data will be required to determine whether the desirable variations in PG38 and PGR, e.g., CS-T04 and CS-T01, point to potentially useful genes for heat tolerance.

### 2.4. Promising CS Lines for Future Studies

Cotton, like some other plant species, produces dynamic responses at the morpho-physiological and cellular levels to tolerate unfavorable temperature and drought conditions. Cotton is cultivated under a wide range of very diverse environments and extremely variable temperatures throughout the world. A recent report showed that increased high temperature and drought stress episodes could reduce crop yields by 50% [73]. Using an index-based classification system for drought-, heat- and combined-resistance, similar to Singh et al. [41], each CS line was categorized as stress tolerance towards heat, drought, and combined heat and drought (Table 3). The heat stress response index (HSRI) varied significantly among the 11 CS lines and ranged from 10.9 (heat-sensitive, CS-T08sh) to 12.4 (heat tolerant, CS-B15sh). Based on the drought stress response index (DSRI), CS-T08sh recorded the lowest value of 9.6, while three CS lines, CS-T07, CS-B15sh, and CS-T18, exhibited a greater value by 14.8% (*p* < 0.05) compared with the drought-sensitive CS-T08sh (Table 3). Based on the cumulative heat and drought stress response index (CHDSRI) value and an increment of SD, four CS lines (CS-T01, CS-T07, CS-B15sh, and CS-T18) were identified as heat- and drought-sensitive (Table 3). Among the tested lines, CS-B07 and CS-T08sh exhibited low drought, low heat, and combined low heat and drought stress tolerance (Table 3). Two lines from *G. tomentosum*, i.e., CS-T07 and CS-T18, showed the highest indices for drought and heat tolerance individually and under a combination of drought and heat tolerance. As CHDSRI had strong positive associations with HSRI (*R*^2^ = 0.97) and DSRI (*R*^2^ = 0.98), CS-T07, CS-T18, and CS-B15sh were identified as the most drought- and heat-tolerant CS lines (Table 3). These CS lines had significantly greater Pn (14%), Gs (29%), T (13%), and PGR (23.6%), as compared to TM-1 under field conditions (Table S1). Future studies should aim towards validating a relationship between total plant variation (cumulative indices) and heat/drought using a diversity panel. The identified heat- and drought-tolerant lines represent a promising means to develop novel climate-ready cotton genotypes after validating under gradient temperature and drought conditions.

### 2.5. Comparative Analysis of CS Lines Physiological and Pollen Phenotype and Modern Advanced Upland Cotton Cultivars

Our comparative analysis among these 11 CS lines and previous observations across 38 cultivars [41] shows that CS lines have increased leaf Pn, Gs, and T by 17%, 23%, and 12% compared to modern advanced Upland cotton cultivars, respectively (Table 1). CS lines have altered the magnitude of these traits. On the other hand, the larger TCHL (25%) and Caro (21%) differences were also observed between the CS lines and modern advanced cotton cultivars at the reproductive stage (Table 1). It was shown that the selection of Pima cotton with higher yield potential and heat resistance was linked with smaller leaf areas [45]. Thus, it could be expected that the decreased leaf characteristic (low SLA) in CS lines could account for genetic differences in gas exchange parameters, yield potential, and heat resistance. The CS lines (CS-T07, CS-B15sh, and CS-T15sh) maintained a considerably greater Gs than the mean for 38 cotton cultivars (0.76 vs. 0.51 mol m^−2^ s^−1^; see [41]), whereas TM-1 exhibited intermediate behavior (Table 1). The difference in CTD between the advanced CS lines and cotton cultivars were observed under field conditions. Averaged across CS lines and cultivars, the CS lines exhibited ~1.3 °C cooler canopy than the advanced Upland cotton cultivars (Table 1). The data show that CMTS in the CS lines is more significant (66%) than the mean of 38 cotton cultivars under field conditions. These findings indicate that the increases in CMTS and lower CTD could be causally related to the increases in Gs in the CS lines. The combination of such trait relationships in cotton showed higher yield potential under hotter climates [45]. Lower CTD (negative values) and higher Gs appear to have provided CS lines with a heat resilient mechanism during pollination, which favored maintaining higher pollen germination under heat stress conditions (38 °C, Table 1). The PGR was comparatively high in CS lines by 19 and 12%, compared to TM-1 and other cotton cultivars, respectively (Table 1). Phenotypic comparison between CS lines and domesticated Upland cotton cultivars made by Singh et al. [41] showed an increased phenotypic magnitude (Table 1).

The available data suggest that these CS lines will be useful for diversifying and enhancing heat and drought tolerance traits in elite Upland cotton. The findings indicate that additional studies are needed to define the physiological potential of CS lines under various combinations of abiotic stress conditions involving high and low temperatures, drought, and nutrient deficit conditions, and various salt conditions. CS line responses to various CO_2_ levels should also be characterized, given that the CO_2_ levels are projected to keep increasing and can affect many of these traits [74,75,76].

### 2.6. Compositional Analysis

Each CS line used in this analysis was bred previously by a pedigreed modified backcross-inbreeding scheme with the maintenance of hypoaneuploid-enforced hemizygosity during the backcross generations and the recovery of a disomic homozygote after self-pollination of the BC5F1 hemizygote. The procedure takes advantage of low-to-moderate transmission of monosomy and segmental monosomy (e.g., segmental deletion heterozygote) via an isogenic hypoaneuploid TM-1-like Upland cotton seed parent and the euploid-only transmission of the donor chromosome via pollen from the backcross hemizygotes. While hemizygous, a chromosome or segment is denied an opportunity for recombination, and in this case, assures that pollen-transmitted donor chromosomes are genetically intact. Meanwhile, recombination and segregation affect the rest of the genome for each generation, i.e., the heterozygous non-target chromosomes and segments. Expectations are that the end-products will be substituted for the targeted chromosome or chromosome segment, plus any donor segments inadvertently retained during backcrossing and rendered homozygous upon selfing and euploid recovery. The presence of donor segments at any location, of course, presents opportunities for the discovery of donor germplasm effects, subsequently refined localization and use in breeding programs. As part of a more extensive single nucleotide polymorphism (SNP) study for all CS lines that will be reported in greater detail separately, we (I) assessed each CS line used in this study for the substitution status of the respective target chromosome or segment with both SSR and SNP markers [77,78], and (II) conducted a genome-wide survey for donor germplasm based on SNP content of the CottonSNP63K array, according to the methods described in previous studies [28,31,77].

Molecular marker analysis confirmed targeted substitutions for 9 of the 11 CS lines, while two of them lacked the intended substitution but contained non-target donor segments (Table 4). As expected, the genomes of all of the CS lines were substituted for donor germplasm at non-target sites varying in size and number (Table 4). These were characterized into two classes, equal to and larger versus smaller than 5 Mbp, and identified as to their chromosomal association (Table 4). Additional details about each donor segment in CS lines will be communicated as part of an upcoming comprehensive analysis of all CS lines. To put relative sizes into perspective, the average *Gossypium* AD-genome (*n* = 26) chromosome is roughly 100 Mbp. The overall genome was flowing cytometrically, estimated to be about 2.4 Gbp [79], about 3500 cM recombinationally [77], and the most recent public sequence assembly is 2.3 Gbp [80]. Thus, each chromosome would, on average, be about 4% of the genome, whereas each 5 Mbp segment would about 0.2% of the genome and about 5% of one chromosome (Table 4).

The presence of moderate numbers of non-targeted alien segments in CS lines is more advantageous than disadvantageous. Still, the detection of their presence underscores the importance of being guarded about the location(s) of genes causing experimentally detected effects. The non-targeted segmental substitutions increase the coverage of the donor genome and thus increase the chances of seeing donor genetic effects of interest. Moreover, the availability of relatively inexpensive high-density genome-wide SNP genotyping as well as very affordable non-destructive simplex genotyping of seed and seedlings makes it relatively simple to track individual segments of hybrids, i.e., after chromosome assortment or homology-based recombination.

### 2.7. Outlook

High temperatures and drought often occur coincidently, and the stresses they cause are often interrelated. Individually or combined, they cause a wide range of phenotype changes that adversely affect cotton development and productivity. How plants deal with heat and drought stresses can be categorized as escape, avoidance, tolerance, and recovery strategies [81]. Drought or heat tolerance provides plants with the ability to endure severe dehydration or heat effects through specific physiological and biochemical activities, such as osmotic adjustment via osmoprotectants [82]. Drought or heat escape is plants’ ability to regulate their growth period or lifecycle, such as the cotton variety with a short life cycle, to get out of the seasonal drought stress or heat period [83]. Drought recovery of plants is a genotype’s capacity to resume growth and yield after exposure to severe drought or heat stresses. It is critical to understand the genetic mechanisms associated with abiotic stresses by detecting the phenotype of the complex cotton morpho-physiological process related to such abiotic stress. We used the indices for heat, drought, and heat and drought combined to identify the CS lines tolerant against such stresses by using a method that was reported in detail in our previous research [35,41,84,85]. It is interesting to note that CS-T07 and CS-T18 showed high tolerance against heat, drought, and heat and drought based on the indices (Table 3). While it remains most likely that the *G. tomentosum* chromosome 7 and 18 substitutions in CS-T07 and CS-T18, respectively, carry the genes responsible for the observed drought and heat tolerance, genes in the other non-targeted donor segments in these lines could be accountable.

It is possible that *G. tomentosum* will be a valuable source of multiple abiotic resilience traits, in that it grows in dry, rocky, or clay coastal areas in the main islands of Hawai at elevations below 390 feet. [86]. Cotton originated in a wild perennial habitat and currently grows in environments that suffer from periodic drought and heat stress [87]. Nonetheless, *G. tomentosum* was reported to be the most heat-resistant genus [27]. Modern cotton elite lines resulting in the subsequent intensive selection are from different latitudes, geographies, and climates to produce high lint yield and improved fiber quality for hand and mechanical harvesting and textile processing. This domestication of elite Upland cotton cultivars has unintentionally narrowed genetic diversity among the cotton lines for several essential traits such as heat tolerance and water use efficiency [88,89]. The primary challenges to the genetic improvement of Upland cotton include (i) low genetic diversity among the growing cultivars, (ii) inadequate information about genomic regions or genes controlling traits such as drought and heat tolerance, and (iii) suitable genetic material for economically important characteristics to improve Upland cotton germplasm. Drought and heat are two of the most critical factors limiting productivity in many cotton-growing areas because cotton is susceptible to heat and water stress [90]. Previous research showed the negative impacts of these stresses on fiber quality and yield [91,92,93]. Identification of heat- and drought-tolerant cotton lines requires correct phenotyping of the associated complex morpho-physiological traits. The innovative method used in this research provided an opportunity to discover several heat- and drought-tolerant cotton CS lines that will be useful in sustainable cotton production against the severe threat of climate crisis from global warming. We advocate future research to estimate genotype-specific anatomical or cellular level tolerance at temporal and spatial scale to better relate total plant variation indices or health with heat and drought stress tolerance.

## 3. Materials and Methods

The overall methods of detecting the stress-resilient cotton lines were based on the stress response indices method used in previous studies [35,41,84,85,94].

### 3.1. Planting Materials

Seeds of eleven CS lines bred for chromosome or chromosome segment substitutions from *G. barbadense* and *G. tomentosum* in the genetic background of *G. hirsutum* (TM-1) were used in the experiment. Each line, e.g., CS-B01, was bred previously by crossing a developed TM-1 hypoaneuploid, e.g., TM-1 monosomic for chromosome-1, as seed parent with the donor species, selecting cytogenetically for the corresponding hypoaneuploid interspecific BC0F1 hybrid; additional introgression was by analogous backcrossing and recovery of hypoaneuploid BCnF1 hybrid, i.e., maintaining the targeted chromosome in a hemizygous state. After the fifth backcross (BC5F1) or higher, the hypoaneuploid BCnF1 was inbred and progeny cytogenetically selected for euploidy to establish a homozygous disomic substitution line, e.g., BC5S1, for seed increase. Five CS lines were bred from crosses with the *G.* *barbadense* donor doubled haploid line 3–79, namely CS-B01, CS-B15sh, CS-B04, CS-B07, and CS-B18, whereas six CS lines were bred from a cross with *G. tomentosum* donor, namely CS-T04, CS-T15sh, CS-T07, CS-T18, CS-T01, and CS-T08sh (Table 1). These CS lines are, as expected, nearly isogenic with each other and TM-1 and differ only for the targeted chromosome or chromosome segment in each line, Upland cotton (*G. hirsutum*). TM-1 is an inbred line bred from the commercial cultivar Deltapine 14 (Delta and Pine Land Co.; Scott, MS, USA) and maintained over 40 generations by self-pollination, as described by Kohel et al. [95]. Comparative analysis of the true-breeding homozygous CS lines with each other and/or TM-1 provides opportunities to associate genetic and trait variations with the lines and substitutions therein. Seeds were sown in an experimental field at the Rodney Foil Plant Science Research Center, Mississippi State University, Mississippi State (lat. 33°28′ N, long. 88°47′ W) during 2010 following a randomized complete block design (RCBD) with four replications. Plots were fertilized with 22.7 kg N pre-plant and 34.0 kg N side-dress 35 days after planting (DAP). The soil type at the Mississippi State University location is a Leeper silt clay loam (fine, smectitic, nonacid, thermic Vertic Epiaquept). Plants were grown under standard production practices for commercial production.

### 3.2. Pollen Viability under Field Conditions and Germination Performance under High-Temperature Stress

Twenty to thirty fresh flowers at anthesis were randomly collected from 10–15 plants for each CS line between 08:00 and 10:00 h from the first fruiting position during flowering, 55 to 60 days after planting. Pollen grains were collected by gently tapping the flowers over Petri dishes that were used to test pollen viability and germination. A pollen viability assay was performed using 2% (aq) 2,3,5-triphenyl tetrazolium chloride (TTC) stain in deionized water, as described by Aslam et al. [96]. The TTC solution stains the viable pollen with a reddish-purple color due to insoluble red formazan formation. A drop of TTC solution was added to the pollen dispersed on a microscope slide. Thirty minutes after staining, the total number of pollen grains and the number of stained pollen grains were counted in two microscopic fields of 2.4 mm^2^ containing more than 100 pollen grains from each microscopic field in all replications.

Taylor’s [97] pollen growth medium, consisting of 2 g agar, 30 g C_12_H_22_O_11_, 5.3 mg KNO_3_, 51.7 mg MnSO_4_, 10.3 mg H_3_BO_3_, 10.3 mg MgSO_4_.7H_2_O made up to 100 mL with deionized water, was used to study pollen germination [19]. The prepared germination medium was poured into three replicate Petri dishes for each CS line and cooled for 15 min for agar solidification. The collected pollen grains were distributed uniformly on the solidified germination medium using a tiny, clean, bristle paintbrush. The Petri dishes were then covered and incubated in an incubator (Precision Instruments, New York, NY, USA) at 30 and 38 °C for 24 h. Each Petri dish per TM-1, CS-line, and temperature treatment combination was considered as a replicate. Total pollen grains and the number of pollen germinated pollen grains were counted using a Nikon SMZ 800 microscope (Nikon Alphaphot YS microscope; Nikon Instrument, Kanagawa, Japan). A pollen grain was considered germinated when the pollen tube length was at least equal to or greater than the grain diameter [94]. Germination percentage was determined as the ratio of the number of germinated pollen grains total number of pollens per field of view. Further, pollen germination response (PGR) was calculated to evaluate the reproductive responses of TM-1 and each CS line using the following equation.

Pollen germination response (PGR) = (PG38 °C/PG30 °C)(1)

where PG38 °C and PG30 °C are the pollen germination percentages of each line at temperatures of 38 and 30 °C, respectively.

### 3.3. Gas Exchange Parameters

Leaf photosynthesis (Pn), transpiration (T), and stomatal conductance (Gs) were measured between 10.00 and 14.00 h on a cloud-free day, using the third or fourth leaves from the top using a portable photosynthesis system (Model LI-6200, LI-COR, Lincoln, NE, USA) 55 to 60 DAP. The measurements were taken at 1500 µmoL photon m^−1^ s^−1^ photosynthetically active radiation (PAR), the chamber temperature was set at 30 °C, and carbon dioxide concentration was maintained at 400 ppm. The internal CO_2_ concentration (Ci) normalized for external CO_2_ concentration (Ca), and the ratio of leaf internal to ambient carbon dioxide concentration (Ci/Ca) were also measured. Instantaneous water-use efficiency (iWUE) was calculated as the ratio between Pn/T, which indicates the amount of net CO_2_ assimilation per unit of water loss.

### 3.4. Leaf Pigment Content and Chlorophyll Stability Index (CSI)

Two sets of leaf samples were collected from five fully expanded leaves during the flowering stage. Five leaf discs, each with an area of 2.0 cm^2^, from each sample, were collected randomly and placed in vials containing four milliliters of dimethyl sulphoxide (DMSO) for chlorophyll (Chl) extraction. The sample vials were incubated at room temperature in the dark for 24 h, the extract decanted into a cuvette, and the absorbance was measured using a Bio-Rad ultraviolet/VIS spectrophotometer (Bio-Rad Laboratories, Hercules, CA, USA) at 470, 648, and 663 nm to calculate concentrations of Chl a, Chl b, and carotenoid content [98]. The total leaf Chl (TCHL) was estimated by summing Chl a and Chl b values.

The chlorophyll stability index (CSI) was assessed according to the procedure described by Murphy (1962). Another set of leaf discs, each with an area of 2.0 cm^2^, was collected similarly from each cotton line and incubated at 56 °C in a temperature-controlled water bath for 1 h. The set of tubes was brought to 25 °C, and the Chl content was measured from the heat-treated samples as described previously. The CSI was estimated as the percentage of TCHL in heated leaf (56 °C) relative to that in fresh leaf using the following equation.

Chlorophyll stability index (CSI, %) = (TCHL in heat-treated)/(TCHL in control) × 100(2)

### 3.5. Leaf Cell Membrane Thermostability (CMTS)

The leaf CMTS in TM-1 and CS-lines was assessed following the procedure described by Singh et al. [41] in each of the three replicates. Two sets (control and treatment) of 10 leaf discs, each disc with a 1.3 cm^2^ area, were cut from five fully expanded third or fourth leaves from the top of the stem axis from each line. The leaf discs were thoroughly rinsed three times with deionized water to remove electrolytes, both adhering to the leaf surface and leaching from the leaf discs’ cut surfaces. All the test tubes with leaf discs were filled with 10 mL of deionized water and capped with aluminum foil to prevent water evaporation. After incubation for 20 min at 55 °C, the sets of test tubes were brought to 25 °C, and initial measurement of the conductance of the control (CEC1) and the treatment (TEC1) was measured by an electrical conductivity meter (Corning Checkmate II; Corning Inc., Corning, NY, USA) at room temperature. Tubes were then autoclaved at 0.1 MPa for 12 min to kill tissues completely, releasing all the electrolytes. Tubes were then cooled to 25 °C, and final conductance was measured (CEC2 and TEC2). Finally, the ratio of the initial to the last electrolyte leakage caused by the elevated temperature was estimated, giving a measure of the extent of damage to cellular membranes using the following equation.

(3) $$\mathrm{CMTS}\left(\%\right)=\frac{\left[1\;-\;\left(\mathrm{TEC}1/\mathrm{TEC}2\right)\right]/\mathrm{Leaf}\;\mathrm{CMT}\;\left(\%\right)\;}{\left[1\;-\;\left(\mathrm{CEC}1/\mathrm{CEC}2\right)\right]}\;\times \;100$$

### 3.6. Canopy Temperature Depression

Canopy temperature depression (CTD) was measured during the reproductive stage between 12:00 and 13:00 h (cloudless, bright days) using a handheld infrared thermometer (Model OS533E-OMEGASCOPE; OMEGA Engineering, Inc., Stamford, CT, USA). Canopy temperature depression was estimated using the equation below

(4) $$\mathrm{Canopy}\;\mathrm{temperature}\;\mathrm{depression}\;\left(CTD,{}^{\circ}C\right)=T_{c}-T_{a}$$

where *T_a_* and *T_c_* refer to the ambient air and canopy temperatures of the target cotton line, respectively.

### 3.7. Specific Leaf Area

Leaf areas of three individual leaves (used for gas exchange) were measured using a leaf area meter (LI-3100: Li-COR, Lincoln, NB, USA). The dry weight and measured leaf area of all lines and cultivar TM1 were used to calculate the specific leaf area (SLA), expressed as cm^2^ g^−1^ d. Wt.

### 3.8. SSR Analysis

The overall method of SSR marker genotyping was as described by Saha et al. [78]. Two to three young leaves from each CS line and TM-1 were used for DNA extraction using DNAeasy cotton genomic DNA extraction protocol (Qiagen kit), and extracted genomic DNAs were checked with Nanodrop and diluted to appropriate concentrations before using for PCR. The PCR-amplifications were performed in a 10 µL reaction mix containing 1 µL 109 PCR buffer, 0.2 µL dNTPs (10 mM each), 1.2 µL 25 mM MgCl_2_, 0.3 µL 5 lM labeled primers (FAM, HEX, VIC, PET), 0.1 µL AmpliTaq Gold DNA polymerase (Applied Biosystems, Foster City, CA, USA), and 5 ng µL^−1^ genomic DNA. With labeled primers, polymorphisms at amplified SSR loci among chromosome substitution lines were detected in denaturing capillary electrophoresis according to manufacturer’s protocol on the Genetic Analyzer 3130xl (Applied Biosystems, Foster City, CA, USA). The size of the amplified products was detected using Gene Mapper 4.0 (Applied Biosystems, Foster City, CA, USA).

### 3.9. SNP Analysis

CS lines were SNP genotyped by (i) extracting DNA using a commercially available kit (Qiagen DNeasy Plant, Macherey-Nagel NucleoSpin Plant II, or OPS SYNERGY Plant DNA), (ii) testing the DNA quality and concentration, (iii) adjusting concentration, and (iv) analyzing with the DNA with the CottonSNP63K Array at the Texas Institute for Genome Sciences and Society (TIGSS) Lab. Genotypes were called with the published cluster file; then, genotypic data were purged of SNPs yielding less-than high uniformity and had more than 10 percent missing data [77]. Linkage groups were identified using a previously published *G. hirsutum* × *G. barbadense* F2 linkage map [77], and SNPs were ordered similarly. Processed genotype data were then scanned to find chromosomes or segments (multiple contiguous marker bins) homozygous or heterozygous for the donor or non-TM1 genotypes. The results were used first to assess if the targeted chromosome or chromosome segment was indeed substituted, and secondarily, to scan the genome for the presence of any inadvertent chromosome or segment substitutions which involved non-targeted chromosomes. The latter donor segment was categorized and tabulated according to physical size: more versus less than 5 Mbp, based on the most recent Upland cotton (TM-1) genome sequence assembly [80].

### 3.10. Stress Response Indices and Statistical Analysis

The indices for heat, drought, and heat and drought combined were developed following the approach that was reported in previous studies [35,41,84,85]. In brief, the relative response index of the CS line was calculated as the value for a CS line divided by the phenotypic value of the Upland recurrent parent, cultivar TM-1. Then, the cumulative indices, namely cumulative heat stress response indices (HSRI), cumulative drought response indices (DRSI), and cumulative heat and drought response indices (CHDSRI) calculated for each line, were used for the classification of high and low stress-tolerant lines. The DSRI was calculated as the summation of the individual relative index of five gas exchange (Pn + Gs + T + iWUE + Ci/Ca), two pigments (TCHL + Caro), two cell and chlorophyll stability indices (CMTS + CSI), and two biophysical (CTD + SLA) traits.

DSRI = (Pn + Gs + T + iWUE + Ci/Ca + TCHL + Caro + CMTS + CSI + CTD + SLA)(5)

Likewise, the HSRI was calculated as the sum of individual relative gas exchange (Pn + Gs + T + iWUE+ Ci/Ca), pigments (TCHL + Caro), cell and chlorophyll stability indices (CMTS + CSI), biophysical (CTD), and reproductive response (PV + PG38/PG30) traits.

HSRI = (Pn + Gs + T + iWUE + Ci/Ca + TCHL + Caro + CMTS + CSI + CTD + PV + PGR)(6)

The CHDSRI of each CS line was calculated as the sum of all 13- traits relative indices used for the phenotyping of heat tolerance and drought tolerance.

CHDSRI = (Pn + Gs + T + iWUE + Ci/Ca + TCHL + Caro + CMTS + CSI + CTD + SLA + PV + PGR)(7)

The 11 CS-lines were classified as low, moderate, and tolerant based on minimum HSRI or DSRI or CHDSI values and an increment of one standard deviation, respectively, as Singh et al. [41] described.

Data were analyzed statistically using ANOVA in RStudio 3.6.1 (https://rstudio.com/) to estimate the significance of variation among lines. Pearson’s correlation coefficient was carried out between all traits using the library (“corrplot”). Dunnett’s test compared the difference between the CS line and TM-1 using the library (DescTools) in RStudio, and a difference detected at *p* < 0.05 by ANOVA was considered significant.

## 4. Conclusions

In summary, replicated analyses of multiple isogenic CS lines at flowering enabled detection of differential responses to various physiological, biophysical, and reproductive traits relevant to performance stability, sustainability, and global warming. The CS lines that imparted significant desirable effects on traits will be useful in breeding programs for targeted introgression of abiotic stress resistance genes of drought and high-temperature conditions in Upland cotton improvement programs, for which additional genetic diversity is needed. For a few traits, the phenotypic extreme(s) among these 11 CS lines exceeded previous observations across 38 Upland cotton cultivars, which suggests that these CS lines could be compelling for diversifying and enhancing these traits in elite Upland cotton. In addition, all or most of the CS lines imparted statistically significant beneficial effects on traits of interest; these lines are likely sources of desirable genes. Upon introgression into elite breeding germplasm lines by marker-assisted breeding, these genes will expectedly extend the phenotypic range of elite cultivars beyond what is currently known. However, there is an urgent need to investigate how cotton cultivars and CS lines will perform under future projected elevated CO_2_ conditions combined with other abiotic stress conditions.

## Figures and Tables

**Table 1:** Leaf gas exchange (Pn—photosynthesis, µmol m^−2^ s^−1^; Gs—stomatal conductance, mol m^−2^ s^−1^; T—transpiration, mmol H_2_O m^−2^ s^−1^, iWUE—instantaneous water use efficiency, mmol CO_2_ mol^−1^ H_2_O; and Ci/Ca—leaf internal to ambient carbon dioxide concentration ratio), pigments (TCHL—total chlorophyll content, µg cm^−2^, and Caro—carotenoids, µg cm^−2^), cell and chlorophyll stability indices (CMTS—cell membrane stability index, %, and CSI—chlorophyll stability index, %), biophysical (CTD—canopy temperature depression, °C; and SLA—specific leaf area, cm^2^ g^−1^ dry weight), and pollen traits (PV—pollen viability, %; PG30—pollen germination at 30 °C; PG38—pollen germination at 38 °C; PGR—pollen germination response) traits of chromosome substitutions (CS) introgressions from *Gossypium barbadense* and *G. tomentosum* into Upland cotton (*G. hirsutum*, cv. TM-1) measured during mid-fruiting at Mississippi State, Mississippi, USA.

CS Line	Pn	Gs	T	iWUE	Ci/Ca	TCHL	Caro	CTD	CMTS	CSI	SLA	PV	PG30	PG38	PGR
CS-B01	33.3	0.51	10.4	3.20	0.71	43.5	9.8	3.40	38.5	92.0	117.0	45.0	42.2	32.0	75.9
CS-B04	38.1	0.70	12.5	3.10	0.73	44.3	9.9	−1.70	48.1	88.0	131.0	50.0	47.2	37.5	79.1
CS-B07	30.8	0.50	10.1	3.10	0.70	42.9	9.3	2.90	35.4	80.0	124.0	52.0	49.3	32.7	66.3
CS-B15sh	37.2	0.83	12.3	3.00	0.71	47.5	10.3	3.50	38.9	82.0	125.0	50.0	47.1	33.9	71.9
CS-B18	34.0	0.56	10.8	3.20	0.71	49.8	11.0	1.40	35.7	87.0	127.0	50.0	46.5	37.2	79.8
CS-T01	33.4	0.63	11.7	2.90	0.74	43.3	9.7	0.90	45.5	83.0	138.0	36.0	33.3	28.0	84.0
CS-T04	32.8	0.47	9.5	3.50	0.68	45.9	10.1	5.00	39.5	85.0	124.0	33.0	29.6	27.0	91.3
CS-T07	38.1	0.86	12.4	3.10	0.71	49.4	10.4	3.70	35.3	86.0	121.0	43.0	40.2	33.2	74.6
CS-T08sh	35.4	0.64	11.5	3.10	0.72	42.7	9.9	−0.70	29.0	94.0	123.0	49.0	45.8	37.8	82.4
CS-T15sh	37.4	0.84	13.3	2.80	0.74	44.6	10.2	−2.50	42.0	90.0	123.0	50.0	46.9	33.1	70.7
CS-T18	37.8	0.76	11.8	3.20	0.67	47.4	10.2	1.90	46.8	83.0	132.0	38.0	34.6	30.4	88.4
TM-1	33.1	0.63	10.7	3.10	0.70	45.2	9.9	4.30	39.8	94.0	129.0	55.0	52.4	33.1	63.3
LSD (*p* = 0.05)	2.9	0.1	1.7	0.28	ns	4.7	ns	3.3	3.8	4.4	6.9	6.5	6.52	5.79	9.7
Significance	***	***	**	**		*		***	***	***	***	***	***	**	***
CS lines mean +	35.3	0.66	11.5	3.11	0.71	45.6	10.1	1.62	39.5	86.4	125.9	45.1	42.1	33.0	78.6
Advanced Upland cotton cultivars ^#^															
Minimum	17	0.17	4.5	1.60	0.43	30.4	6.7	1.2	15.6	72	155.9	43.8	25.6	15.4	42.8
Maximum	38.6	0.85	14.4	4.40	0.84	38.1	10.2	4.3	40.4	92.9	199.6	66.3	49.9	41.8	94.4
Mean	29.3	0.51	10.1	3.20	0.67	34.0	8.00	2.9	26.1	82.1	184	60.2	40.1	27.7	68.9

+ mean of 11 CS lines. ^#^ Phenotypic data of 38 diverse Upland cotton cultivars were obtained from an earlier field study [41] in order to compare results to those for the 11 CS lines. * *p* < 0.05, ** *p* < 0.01, *** *p* < 0.001, ns-nonsignificant.

**Table 2:** Percent absolute difference in trait means between selected CS lines and TM-01 where a significant difference was detected based on Dunnett’s test (*p* < 0.05) for leaf photosynthesis (Pn), leaf stomatal conductance (Gs), leaf transpiration (T), the ratio of Pn to T (iWUE), the cell membrane stability index (CMTS), the chlorophyll stability index (CSI), canopy temperature depression (CTD), specific leaf area (SLA), pollen viability (PV), pollen germination at 30 °C (PG30), and the pollen germination response (PGR) measured during mid-fruiting.

CS Line	Pn	Gs	T	iWUE	CTD	CMTS	CSI	SLA	PV	PG30	PGR
CS-B01								−11.9	−10.2	−10.2	+12.6
CS-B04	+4.9				−6.0	+8.3					+15.8
CS-B07							−13.6				
CS-B15sh	+4.1	+0.20					−11.3				+8.6
CS-B18							−7.2				+16.5
CS-T01						+5.7	−11.1		−19.0	−19.0	+20.6
CS-T04				+0.42			−9.0		−22.8	−22.8	+27.9
CS-T07	+4.9	+0.23					−7.6		−12.1	−12.1	+11.3
CS-T08sh					−5.0	−10.9					+19.1
CS-T15sh	+4.2	+0.21	+2.60		−6.8						
CS-T18	+4.6					+6.9	−10.6		−17.8	−17.8	+25.1

Dunnett’s test was performed on trait means to examine differences between each of the CS lines and TM-01. +, significant positive performance; −, significant negative performance compared with TM-1.

**Table 3:** Classification of CS lines (chromosome-specific introgressions from *G. barbadense* and *G. tomentosum* into Upland cotton (*G. hirsutum*, cv. TM-1)) into drought-, heat- and combined heat and drought-tolerant groups based on the heat and drought cumulative relative response index.

Heat Tolerant			Drought Tolerant			Combined Heat and Drought Tolerant		
Low	Moderate	High	Low	Moderate	High	Low	Moderate	High
CS-T01 (11.2)	TM-1 (12)	CS-B15sh (12.4)	CS-B04 (10.2)	CS-T04 (10.8)	CS-T07 (11.4)	CS-T01 (12.3)	CS-T04 (12.8)	CS-T07 (13.4)
CS-T15sh (11.1)	CS-T04 (11.9)	CS-T07 (12.4)	CS-T15sh (10.1)	CS-B01 (10.4)	CS-B15sh (11.3)	CS-T15sh (12.1)	CS-B18 (12.5)	CS-B15sh (13.4)
CS-B07 (11)	CS-B01 (11.5)	CS-T18 (12.1)	CS-B07 (9.9)	CS-B18 (10.3)	CS-T18 (11.1)	CS-B07 (11.9)	CS-B01 (12.4)	CS-T18 (13.1)
CS-T08sh (10.9)	CS-B18 (11.5)		CS-T08sh (9.6)	CS-T01 (10.3)	TM-1 (11)	CS-T08sh (11.8)	CS-B04 (12.4)	TM-1 (13)
	CS-B04 (11.4)							

The relative performance index of chromosome substitution (CS) lines was calculated as the value for a CS line divided by the phenotypic value of TM-1. The drought stress response index (DSRI) is the sum of the individual relative index of five gas exchange (Pn + Gs + T + iWUE + Ci/Ca), two pigments (TCHL + Caro), two cell and chlorophyll stability indices (CMTS + CSI), and two biophysical (CTD + SLA) traits. The heat stress response index (HSRI) is the sum of individual gas exchange (Pn + Gs + T + iWUE + Ci/Ca), pigments (TCHL + Caro), cell and chlorophyll stability indices (CMTS + CSI), biophysical (CTD), and pollen vitality response (PV + PG38/PG30) traits. The combined heat and drought stress response index (CHDSRI) is the sum of all 13 individual response indexes (see Materials and methods). The different CS lines were classified as low, moderate, and tolerant based on minimum HSRI or DSRI or CHDSI values, and an increment of one standard deviation, respectively, described by Singh et al. [41].

**Table 4:** Classification of 11 CS lines (chromosome-specific introgressions from *G. barbadense* and *G. tomentosum* into Upland cotton (*G. hirsutum*, cv. TM-1)) into drought-, heat- and heat and drought-tolerant groups based on relative stress response index.

CS Line	SSR Markers *	Non Target (>5 Mb) **	Non Target (<5 Mb) ***	Target ****
CS-B01	BNL2921-158, BNL3580-207, BNL3848-197, BNL-3888-197, CIR009-233, NAU2437-235	c06, c25	c09, c25	Substituted
CS-B04	BNL2821 = 192, BNL3089-124, BNL3988-120, CIR222-276, CIR249-190	-	c18; c20	Substituted
CS-B07	-	c11, c16,	c04, c16, c20, c21, c22	TM-1
CS-B15sh	-	c07	c02, c10; c13, c20	TM-1
CS-B18	BNL0193-111, BNL3479-251, CIR216-141, Gh501-201, TMB2762-205,	-	c12, c16, c17, c19, c24, c25, c26	Substituted
CS-T01	BNL1693-244, BNL2921-162, BNL3580 = 202, BNL3848-223, BNL3888-182, BNL2437-247	c25	c03, c07, c10, c20, c25	Substituted
CS-T04	-	-	c18	Substituted
CS-T07	BNL1395-163, BNL1597-228, CIR141-170, CIR169-135, Gh548-113,	c02, c21	c05, c08	Substituted
CS-T08sh	-	c12, c20	c05, c06, c09, c13, c21, c25	Substituted
CS-T15sh	-	c09, c12	c08, c14, c17	Substituted
CS-T18	BNL0193-113, BNL1721-171, BNL243-135, BNL3479-244, BNL569-142, CIR216-141, CIR216-141	c01	c26	Substituted

* The marker name- marker size in bp [78]; ** single nucleotide polymorphism (SNP) markers associated with more than 5 Mb size [77]; *** SNP markers associated with less than 5 Mb size [77]; and **** confirmation on the cytological and molecular evidence concerning the substituted chromosome or chromosome segment.

## Supplementary Materials

The following are available online at https://www.mdpi.com/2223-7747/9/12/1747/s1, Table S1: The relative performance of cotton chromosome substitution (CS) lines compared with parent Upland cultivar, TM-1.

## Abbreviations

Car	Carotenoids
Ci/Ca	Ratio of leaf internal to ambient carbon dioxide concentration
CMTS	Cell membrane thermostability
CSI	Chlorophyll stability index
CTD	Canopy temperature depression
DSRI	Drought stress response index
Gs	Stomatal conductance
HSRI	Heat stress response index
iWUE	Instantaneous leaf water use efficiency
PG	Pollen germination
PGR	Pollen germination response
Pn	Leaf net photosynthesis
PV	Pollen viability
SLA	Specific leaf area
SNP	Single nucleotide polymorphism
SSR	Simple sequence repeat
TCHL	Total leaf chlorophyll
T	Transpiration
