# Supplementary material for: High-Temperature and Drought-Resilience Traits among Interspecific Chromosome Substitution Lines for Genetic Improvement of Upland Cotton
Source: Plants (Basel). 2020 Dec 10;9(12):1747. doi: 10.3390/plants9121747 (PMC7763690; doi:10.3390/plants9121747)
Supplement: Supplementary file 1 [file plants-09-01747-s001.pdf]

Table S1. The relative performance of cotton chromosome substitution (CS) lines compared with parent Upland cultivar, TM-1. The traits were leaf gas exchange (Pn – photosynthesis, μmol m^{-2} s^{-1}; Gs - stomatal conductance, mol m^{-2} s^{-1}; T - transpiration, mmol H_2O m^{-2} s^{-1}, and iWUE – *instantaneous* water use efficiency, mmol CO_2 mol^{-1} H_2O; and Ci/Ca - leaf internal to ambient carbon dioxide concentration ratio), pigments (TCHL - total chlorophyll content, μg cm^{-2}, and Caro – carotenoids, μg cm^{-2}), cell and chlorophyll stability indices (CMTS - cell membrane stability index, %, and CSI- chlorophyll stability index, %), biophysical (CTD - canopy temperature depression, °C; and SLA - specific leaf area, cm^2 g^{-1} dry weight), and reproductive (PV - pollen viability, %; PG30 - pollen germination at 30°C; PG38 - pollen germination at 38°C; PGR- pollen germination response) of chromosome-specific introgressions from *Gossypium barbadense* and *G. tomentosum* into Upland cotton (*G. hirsutum*, cv. TM-1) measured during mid-fruiting at Mississippi State, Mississippi, USA.

Line	Pn	Gs	T	iWUE	Ci/Ca	TCHL	CTD	Caro	CMTS	CSI	SLA	PV	PG30	PG38	PGR
CS-B01	0.60	-19.44	-2.82	3.35	1.13	-3.56	-20.51	-0.79	-3.24	-2.22	-9.28	-18.37	-19.42	-3.29	19.88
CS-B04	14.80	11.26	16.87	-0.77	4.25	-1.88	-140.17	0.26	20.83	-5.80	2.12	-9.37	-9.91	13.09	24.97
CS-B07	-7.15	-20.69	-6.29	-0.99	-0.43	-4.94	-33.33	-6.25	-11.20	-14.56	-3.40	-5.49	-5.80	-1.18	4.65
CS-B15sh	12.31	31.32	14.42	-1.70	1.31	5.27	-18.80	4.72	-2.30	-12.04	-2.71	-9.49	-10.03	2.19	13.51
CS-B18	2.68	-11.26	0.31	2.27	1.32	10.19	-67.52	11.11	-10.46	-7.72	-1.09	-10.51	-11.11	12.18	26.05
CS-T01	0.79	-0.94	8.90	-7.00	4.77	-4.04	-78.63	-1.67	14.27	-11.89	7.05	-34.34	-36.31	-15.53	32.56
CS-T04	-1.09	-24.96	-11.60	13.52	-4.16	1.69	17.95	2.82	-0.72	-9.60	-3.64	-41.11	-43.47	-18.47	44.10
CS-T07	14.80	36.06	15.64	-0.89	0.83	9.50	-12.82	5.40	-11.46	-8.09	-5.51	-21.91	-23.17	0.26	17.80
CS-T08sh	6.85	1.46	7.06	-0.38	2.71	-5.44	-117.09	0.21	-27.25	0.09	-4.44	-11.88	-12.56	13.99	30.09
CS-T15sh	12.81	32.88	24.23	-9.33	5.61	-1.14	-158.12	3.53	5.49	-4.32	-4.07	-9.85	-10.42	0.02	11.58
CS-T18	14.00	20.11	10.06	3.70	-4.85	4.95	-56.41	3.66	17.36	-11.33	2.79	-32.14	-33.98	-8.19	39.59

The relative performance of a CS line, describing the deviation from TM-1 in percentage. + = positive relative performance; – = negative relative performance compared with TM-1.
